# Effect of *Saengmaeksan* on Fatigue, Liver Function, and Immunity Combined with High-Intensity Training

**DOI:** 10.1155/2023/3269293

**Published:** 2023-06-30

**Authors:** Shi Juan, Jae-Hoon Lee, Se-Jong Won, SiYeon Oh, Min-Seong Ha

**Affiliations:** ^1^Xi'an FanYi University, Shaanxi 710105, China; ^2^Department of Sports Science, College of the Arts and Sports, University of Seoul, 163 Seoulsiripdaero Dongdaemun-gu, Seoul 02504, Republic of Korea; ^3^Taekwondo Diplomacy, Chungbuk National University of Health Science, 10 Deogam-gil Naesu-eup Cheongwon-gu, Cheongju-si 28644, Chungcheongbuk-do, Republic of Korea; ^4^College of Art and Physical Education, Chosun University, 309 Pilmun-daero Dong-gu, Gwangju 61452, Republic of Korea

## Abstract

*Saengmaeksan* (SMS) is a traditional drink that consists of three natural herbs, *Lirio platyphlla*, *Panax ginseng*, and *Schisandra chinensis*, and improves fatigue, liver function, and immunity. Moderate-intensity exercise has a positive effect on fatigue, liver function, and immune function, whereas long-term high-intensity training has a negative effect on these aspects. We hypothesized that SMS intake will improve fatigue (ammonia, lactic acid), liver function (aspartate transaminidase (AST) and alanine aminotransferase (ALT)), and immunity (IgA, IgG, IgM) with high-intensity training. To investigate this hypothesis, 17 male college tennis players were randomly assigned to SMS and placebo groups with high-intensity training. SMS and placebo were consumed in 110 mL doses for a total of 770 mL. High-intensity training was performed at 70%–90% of heart rate reserve, conducted five times weekly for 4 weeks. A significant interaction effect was observed between the SMS and control (CON) groups regarding ammonia, ALT, and IgA levels. Ammonia levels significantly decreased in the SMS group, but there was no difference in the lactic acid levels. AST significantly decreased in the SMS group. IgA significantly increased in the SMS group, IgM significantly decreased in both groups, but there was no change in IgG. The correlation analysis revealed positive correlation in *Δ*AST vs. *Δ*ALT, *Δ*ALT vs. *Δ*IgG, and *Δ*IgA vs. *Δ*IgG in the SMS group. These findings demonstrate that SMS intake can reduce ammonia, AST, ALT, and IgM levels, while causing an increase in IgA, which has a positive effect on fatigue reduction, liver function, and immunoglobulins in a high-intensity training or related environment.

## 1. Introduction

Fatigue research focuses on the performance of athletes who require high performance. Fatigue is caused by the accumulation of lactic acid and ammonia in the blood during exercise, resulting in a negative correlation with exercise ability [1]. Ammonia is a direct precursor leading to the production of other metabolites that trigger fatigue during strenuous exercise. Elevated blood ammonia levels are associated with various functional and metabolic neurological disorders, as well as fatigue [2]. Blood ammonia and lactate levels are elevated in well-trained athletes that perform high-intensity exercise compared to those of untrained individuals. Thus, the metabolic function of ammonia is essential for athletes to maintain performance [3].

High-intensity exercise also affects liver function. Exercise increases blood flow to the skeletal muscles while decreasing blood flow to the liver, which can lead to liver damage. Intense exercise releases enzymes from the liver, raising indicators related to liver damage such as aspartate aminotransferase (AST) and alanine aminotransferase (ALT); thus, continuing at such intensity for long periods of time can cause chronic liver damage [4]. An increase in AST alone may indicate heart or muscle disease, and abnormal elevation of both AST and ALT can increase liver-related mortality [5]. In an animal experiment investigating the effects of high-intensity exercise on the liver in mice, exercise at 75% and 90% maximal oxygen consumption (VO_2_max) increased both AST and ALT levels. A higher exercise intensity was associated with more distinct edema and inflammatory response in the liver [6]. In a human experiment, exercise-induced oxidative stress and skeletal muscle damage increased AST and ALT levels, similar to the effects found in animal experiments [7].

Serum immunoglobulin (Ig) is an immune status indicator. Low Ig levels indicate immunodeficiency, whereas high Ig levels may cause liver, chronic inflammatory, and blood diseases [8, 9]. Vigorous exercise induces an increase in inflammatory cytokine levels. For example, marathon races increase interleukin (IL)-6 levels by a factor of 100 and also show a high correlation with Ig levels [10, 11]. In addition, endurance athletes have an increased incidence of upper respiratory tract infections after high-intensity training. Because the concentration of IgA appears to be low after high-intensity exercise, the levels of Igs can help determine the risk of infection and the degree of training in athletes [12].

Numerous researchers have made greater efforts to find a way to maintain athletic performance while maintaining high training intensities. Several studies have been conducted on the acute effects of fatigue substances, liver function, and immune function [13–15]. However, longitudinal studies to confirm the long-term effects of consuming natural sports drinks are limited.

With growing interest in traditional Chinese medicine tea beverages, the U.S. National Institutes of Health has defined herbal and botanical supplements containing herbs as “health supplements” [16]. These beverages have synergistic effects, if prepared properly by a combination of one or more herbal formulas [17]. SMS is a traditional herbal beverage prepared by blending three natural medicinal herbs, *Lirio platyphlla*, ginseng, and *Schisandra chinensis*, and is often used for rejuvenation during the summer [18].

Various studies have recorded the health effects of SMS, such as a reduction in body fat, increase in exercise time, decrease in heart rate [19], an antihyperuricemic effect [20], and an antioxidant effect [21]. These effects can be predicted according to the individual effects of the component substances in SMS. *L. platyphylla*, which accounts for half of SMS, produces steroid saponin as its main metabolite, followed by flavonoids and phenols [22]. Saponins are mainly produced in Liliaceae plants and induce various biochemical changes such as improving cholesterol levels by regulating lipids and oxidative stress [23]. Additionally, various pharmacological effects such as immunomodulatory, anticancer, and antiviral effects [24] have been verified. More than 20 types of ginsenosides, commonly referred to as steroidal saponins and triterpene saponins, constitute the main components of ginseng [25]. The effects of ginseng include central nervous system stimulation and inhibition, neurotransmitter control, memory deficit prevention, anticancer activity, and immunomodulation [26]. Finally, *S. chinensis*, which produces the five-flavor fruit or magnolia berry, has more than 40 beneficial components. Among them, lignans are the main active ingredients with antioxidant effects, liver protection, immune function improvement, osteoporosis improvement, and antidepressant effects [27]. Therefore, we hypothesized that SMS intake will improve fatigue, liver function, and immunity under high-intensity training, which was tested in a placebo-controlled study of male college tennis players.

## 2. Materials and Methods

### 2.1. Participants

The sample size of 18 participants was calculated using G^*∗*^Power 3.1 (effect size: 0.25, significance: 0.05, power: 0.5). Therefore, we recruited a total of 20 participants considering potential dropouts. Before participating, the purpose and procedure of the study were fully explained to the participants. All participants provided written informed consent approved by the Institutional Human Research Committee, which followed the Declaration of Helsinki and Ethical Principles. The participants were also provided financial compensation for the research intervention. The study participants were male tennis players, 20–30 years of age, at Pusan National University. After confirming that there were no problems with SMS intake, the participants were randomly assigned to the SMS and the placebo control (CON) groups. Data from the SMS group (*n* = 9) and the CON group (*n* = 9), disregarding two dropouts, were included in the final analysis. The study participant characteristics are shown in Table 1.

### 2.2. Study Design

**Figure 1:**
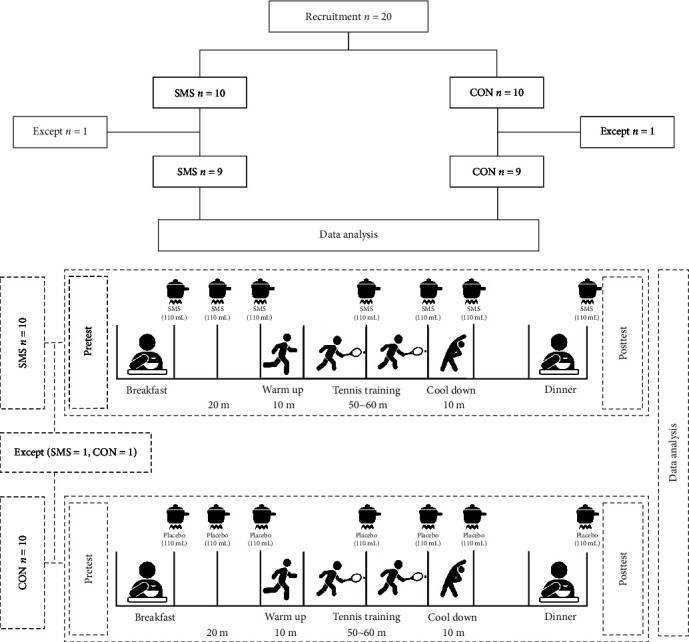
Study design. Twenty participants, excluding dropouts, were randomly assigned to either the SMS (*n* = 9) or placebo (*n* = 8) group, consuming 100 mL doses at a time, seven times (770 mL total) daily. High-intensity training was conducted five times weekly for 4 weeks, and training intensity was performed at 70%–90% of the heart rate reserve. The following outcomes were measured after 4 weeks (posttest): ammonia, lactic acid, AST, ALT, IgA, IgG, and IgM. SMS, *Saengmaeksan*; CON, control.

This study was set as a randomized controlled trial to confirm the effect of SMS intake for 4 weeks. The study was double-blinded to increase the data reliability. Study participants were instructed not to exercise excessively the day before the measurements. A basic medical questionnaire was issued to assess whether the participants had high blood pressure, heart disease, or musculoskeletal disease and were receiving drug treatment, or whether there were any restrictions on participation in the study. The overall design of this study is presented in Figure 1. The timing of SMS intake and exercise program structures were established with reference to the study of Kwak et al. [28]. The placebo was developed to have the same color and shape as SMS and was also provided in the same packaging to maintain blinding for the participants and investigators. A total of 770 mL SMS was ingested seven times daily (110 mL per dose); after breakfast, 20 min before exercise, immediately before the start of exercise, immediately after the main exercise, 30 min after the main exercise, after the type of exercise program, and after dinner. The exercise program was conducted five times weekly for 4 weeks with a length of 80–90 min per session. The program consisted of a 10 min warmup (stretching and running), 40–50 min of the main exercise (stroke, volley, and game), and 10 min of cooldown (stretching and running). During the main exercise, high-intensity exercise was performed at an intensity of 70%–90% of the spare heart rate (rated perceived exertion (RPE) [3–7]). Exercise intensity was confirmed using a heart rate monitor watch (Polar RS400sd, APAC, 90026360; Polar, NY, USA). For a comprehensive evaluation, the researcher monitored all participants during the intervention period, and the CON group was particularly encouraged to maintain their usual lifestyle.

### 2.3. SMS Preparation

*L*. *platyphlla* (15 g), *P. ginseng* (7.5 g), and *S. chinensis* (7.5 g) extracts were mixed (at a ratio of 2 : 1 : 1) with 1,500 mL water using an herbal extractor. The mixed solution was then heated to 100°C under 0.7 kg/cm^2^ pressure for 3 hr and extracted. The extract was sealed and stored in a small plastic bag [28].

### 2.4. Blood Sampling

Blood samples were taken twice before and after exercise to analyze the measurement variables. All study participants were asked to fast for at least 10 hr before measurements. Between 8 and 9 AM, 10 mL of blood was collected from the forearm vein. The collected blood was placed in an ethylenediaminetetraacetic acid-treated tube to inhibit coagulation. The serum was then separated by centrifugation at 3,000 rpm for 10 min (Combi-514, Hanil, Korea).

Ammonia was analyzed using the NH3L system (Roche, Switzerland), and lactic acid was measured using a Cobas Integra 800 (Roche, Switzerland) biochemical analyzer. Liver function (AST and ALT levels) was analyzed using AST-ALT reagents (Bayer, USA) and an ADVIA 1650 device (Bayer, Japan). IgA, IgG, and IgM levels were analyzed by immunoturbidity measurements using IGGT, IGA, and IGM devices (Roche Diagnostic System, Germany). After mixing each antiserum with serum and incubation at 37°C for 10 min, absorbance was measured at 340 nm (Integra 800, Roche, Switzerland), and the concentration of IgA, IgG, and IgM was determined from the concentration in the standard curve [29].

### 2.5. Statistical Analysis

All data were analyzed using IBM SPSS 27.0. The data are presented as the mean and standard deviation. Two-way analysis of variance was performed with treatment (SMS and placebo) and time (before exercise and after exercise) as independent variables to investigate the effect of SMS intake on fatigue substances, liver function, and immune function during high-intensity exercise. The least-significant difference (LSD) test was used for post hoc analysis. Pearson's correlation analysis was performed using the difference values (*Δ*, delta) measured before and after exercise to confirm the relevance of the differences in the responses of the variables after treatment. The significance level was set as *p* < 0.05.

## 3. Results

### 3.1. Effects of SMS Intake on Fatigue-Related Substances during High-Intensity Exercise

**Figure 2:**
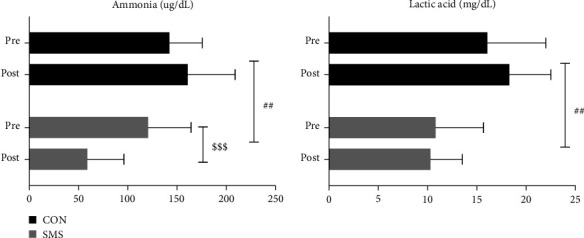
Effect of *Saengmaeksan* intake on fatigue-related substances in tennis players. Significant differences were observed between the SMS and CON groups in the levels of ammonia (time effect, *p* < 0.05; group effect, *p* < 0.01; interaction effect, *p* < 0.001). Ammonia levels were significantly decreased in the SMS group (*p* < 0.001). Significant differences were observed between SMS and CON groups in the levels of lactic acid (group effect: *p* < 0.01). All data are presented mean ± SD. ^$$$^*p* < 0.001 vs. pre and ^##^*p* < 0.01 vs. group.

Figure 2 shows the effect of SMS intake on fatigue-related substance levels after 4 weeks of high-intensity exercise. Ammonia levels decreased from 120.56 ± 43.61 *μ*g/dL to 58.89 ± 37.21 *μ*g/dL in the SMS intake group and increased from 142.22 ± 35.32 *μ*g/dL to 160.78 ± 48.18 *μ*g/dL in the CON group. There were significant main effects of time (*F* = 6.244, *p* < 0.05) and treatment (*F* = 12.455, *p* < 0.01), and a significant time × treatment interaction (*F* = 21.620, *p* < 0.001). The lactic acid level decreased from 10.81 ± 88 mg/dL to 10.33 ± 3.23 mg/dL in the SMS group and from 18.30 ± 4.22 mg/dL to 16.06 ± 5.94 mg/dL in the CON group. There was a significant effect of treatment (*F* = 11.486, *p* < 0.01) but no significant effect in the time × treatment interaction. The post hoc analysis indicated that only the SMS intake group showed a significant change only in ammonia levels among the fatigue-related substance variables (*p* < 0.001).

### 3.2. Effects of SMS Intake on Liver Function during High-Intensity Exercise

**Figure 3:**
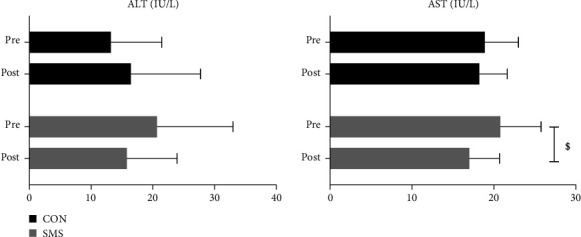
The effects of *Saengmaeksan* intake on liver function in tennis players. Significant differences were observed between the SMS and CON groups in the ALT levels (interaction effect: *p* < 0.05). AST levels were significantly decreased in the SMS group (*p* < 0.05). All data are presented as mean ± SD. ^$^*p* < 0.05 vs. pre.

Figure 3 shows the effect of SMS intake on liver function after 4 weeks of high-intensity exercise. The ALT level decreased from 20.67 ± 12.32 IU/L to 15.78 ± 8.14 IU/L in the SMS intake group and increased from 13.22 ± 8.21 IU/L to 16.44 ± 11.29 IU/L in the CON group. There was a significant effect of the time × treatment interaction (*F* = 4.963, *p* < 0.041). The AST level decreased from 20.78 ± 4.97 IU/L to 17.00 ± 3.67 IU/L in the SMS intake group and from 18.89 ± 4.11 IU/L to 18.22 ± 3.42 IU/L in the CON group. However, there was no statistically significant effect found. The post hoc analysis indicated that the SMS intake group only showed a significant difference in AST among the liver function variables (*p* < 0.05).

### 3.3. Effects of SMS Intake on Immune Function during High-Intensity Exercise

**Figure 4:**
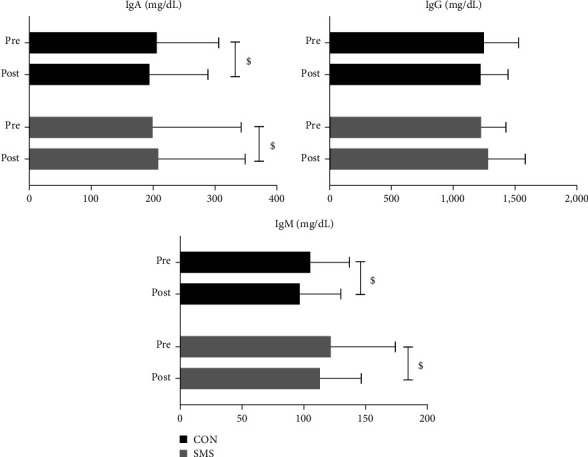
Effect of *Saengmaeksan* intake on immunoglobulins in tennis players. Significant differences were observed between the SMS and CON groups in the levels of IgA (interaction effect: *p* < 0.01). IgA levels were significantly increased in the SMS group (*p* < 0.05) but were significantly decreased in the CON group (*p* < 0.05). No significant differences were observed between SMS and CON groups in the levels of IgG. Significant differences were observed between groups in the levels of IgA (time effect:  ^*∗∗∗*^*p* < 0.01). IgM levels were significantly decreased in the SMS group (*p* < 0.05) and significantly decreased in the CON group (*p* < 0.05). All data are presented mean ± SD. ^$^*p* < 0.05 vs. pre.

Figure 4 shows the effect of SMS intake on immune function after 4 weeks of high-intensity exercise. The IgA level increased from 199.00 ± 143.22 mg/dL to 208.33 ± 140.54 mg/dL in the SMS group and decreased from 205.78 ± 100.27 mg/dL to 194.11 ± 94.42 mg/dL in the CON group. There was a significant effect of the time × treatment interaction (*F* = 13.024, *p* < 0.01). The IgG level increased from 1,224.56 ± 201.71 mg/dL to 1,281.59 ± 300.22 mg/dL in the SMS group and decreased from 1,247.56 ± 279.40 mg/dL to 1,220.89 ± 221.55 mg/dL in the CON group. However, there was no statistically significant effect observed. The IgM level decreased from 121.67 ± 52.51 mg/dL to 113.11 ± 33.41 mg/dL in the SMS ingestion group and decreased from 105.22 ± 31.78 mg/dL to 96.67 ± 33.41 mg/dL in the CON group. There was a significant effect only for time (*F* = 15.271, *p* < 0.001). The post hoc analysis indicated that among the variables of immune function, there was a statistically significant increase in IgA in the SMS intake group (*p* < 0.05) and a statistically significant decrease in the CON group (*p* < 0.05). However, there was a statistically significant decrease in IgM in both the SMS intake and CON groups (*p* < 0.05).

### 3.4. Correlation between Changes in Measurement Variables

**Figure 5:**
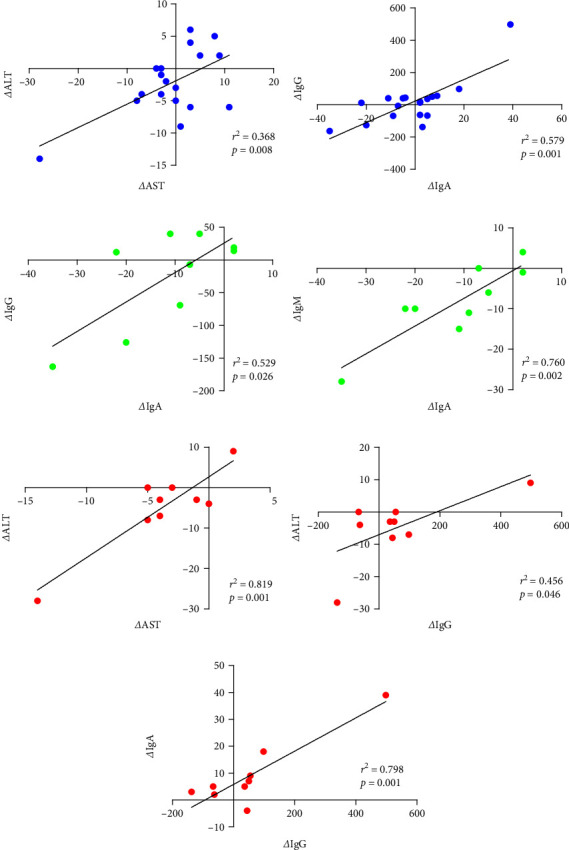
Correlation of changes in fatigue-related substances, liver function variables, and immunoglobulins. All data are significantly positively correlated. (a, b) *Δ*ALT vs. *Δ*AST and *Δ*IgG vs. *Δ*IgA (blue). (c, d) *Δ*IgA vs. *Δ*IgG and *Δ*IgA vs. *Δ*IgM in the CON group (green). (e–g) *Δ*ALT vs. *Δ*AST, *Δ*ALT vs. *Δ*IgG, and *Δ*IgA vs. *Δ*IgG in the SMS group (red).

Figure 5 shows the results of Pearson's correlation analysis between changes in each variable according to SMS intake after 4 weeks of high-intensity exercise. There was a positive correlation between *Δ*IgA and *Δ*IgG (*r* = 0.761, *p* = 0.001), followed by *Δ*ALT and *Δ*AST (*r* = 0.607, *p* = 0.008). The correlation analysis between changes by group indicated that the CON group had a positive correlation with *Δ*IgA vs. *Δ*IgM (*r* = 0.872, *p* = 0.002), followed by *Δ*IgA vs. *Δ*IgG (*r* = 0.727, *p* = 0.026). The SMS group had a positive correlation with *Δ*ALT vs. *Δ*AST (*r* = 0.905, *p* = 0.001), followed by *Δ*IgA vs. *Δ*IgG (*r* = 0.893, *p* = 0.001), and *Δ*ALT vs. *Δ*IgG (*r* = 0.675, *p* = 0.046).

## 4. Discussion

We confirmed the correlation between fatigue (ammonia and lactic acid), liver function (ALT and AST), and immunity (IgA, IgG, and IgM) variables by analyzing changes between treatments (SMS or placebo control), time, and time × treatment interaction to determine whether SMS intake can protect and/or improve the negative effects of high-intensity exercise. We found a significant interaction effect among ammonia, ALT, and IgA, and a positive correlation between *Δ*ALT and *Δ*AST and between *Δ*IgA and *Δ*IgG. However, these results suggest that SMS intake during high-intensity exercise can have a positive effect on fatigue and liver function, and that there is a positive correlation between changes in liver and immune function variables.

In a meta-analysis examining the effect of ginseng on fatigue, consumption of 1,000 mg or more per day resulted in a decrease in fatigue, although the number of included studies was small [30]. In this study, approximately 3,500 mg of ginseng was consumed by ingesting 770 mL of SMS daily. We believe that the *L. platyphylla* and *S. chinensis* components caused a significant decrease in ammonia. In addition, the reduction of AST and ALT helped to reduce the ammonia level since the improvement of fatigue-related substances found in this study has detoxification and ammonia removal functions owing to the notable roles of SMS on liver function [2].

Continuous muscle contraction due to high-intensity training causes skeletal muscle fatigue. Therefore, lactic acid transport and recovery are critical because the accumulation of high concentrations of lactic acid inhibits the release of calcium from the sarcoplasmic reticulum and interferes with muscle contraction [31]. No specific changes were found in lactic acid in this study. However, well-trained athletes show higher ammonia levels during exercise than untrained athletes [3]. Therefore, the results of this study regarding the effect of ammonia level reduction and the time × treatment interaction are quite meaningful.

There is a positive correlation between the increase in blood lactate and ammonia during exercise [32]. However, no such relationship was found in the recovery of lactic acid and ammonia after maximal exercise in elite athletes [33]. We did not identify a significant relationship between ammonia and lactic acid changes in the present study; there was a significant decrease in ammonia in the SMS group but no change in lactic acid. These results suggest that lactate and ammonia in the blood at rest can be regulated through different mechanisms. Importantly, we found that ammonia can be reduced through SMS intake, although there is no effect on the change of lactic acid.

*S. chinensis* has a long history of use in traditional Chinese medicine with various pharmacologically significant roles, such as an antioxidant, antitumor promotion agent, and calcium antagonist [27]. It is particularly effective for liver protection because the main components are lignans and polysaccharides [34]. High-intensity exercise can increase AST and ALT levels by causing oxidative stress and muscle damage [7]. When the liver is damaged, ALT is released into the bloodstream and levels increase. Because an increase in AST levels in blood at a low level conveys damage to the heart or muscle, it is essential to protect the liver in athletes [5].

ALT showed a significant time × treatment interaction in the SMS group. The significant decrease in AST compared to that before exercise is attributed to the removal of free radicals and a reduction in oxidative stress by the *S. chinensis* component of SMS, thereby reducing liver damage [35]. Although ALT is mainly found in the liver, it can also be found in the heart, skeletal muscle, and kidneys. Therefore, a significant decrease in AST is accompanied by improvements to the liver, heart, skeletal muscle, and kidneys, indicating that SMS intake can induce various benefits [5].

Ginseng is a natural ingredient often used in oriental medicine to improve fatigue and stamina. Various effects of ginsenoside, the main component of ginseng, have been reported on the central nervous system, neuroendocrine function, immune function, and cardiovascular system, depending on the dosage and method [36]. Ginseng extracts positively affect the anti-inflammatory system, intestinal microbiome, and immune system in rats. In this study, the cytokine IL-10 and IgA levels were increased, while IgG and IgM levels decreased [37]. The induction of IL-10 protects against intestinal and systemic inflammatory responses by limiting the development of proinflammatory Th17 cells [38]. IgA significantly increased in the SMS group with a significant time × treatment effect in post hoc analysis. In addition, the post hoc analysis indicated that SMS intake induced a significant decrease in IgM.

Regular exercise can strengthen the immune system. However, strenuous physical activity can cause stress and weaken the immune system, which increases the risk of respiratory diseases in athletes who engage in high-intensity endurance exercise [39, 40]. A reduction in IgA levels was commonly found among cross-country athletes, kayakers, female hockey players, and swimmers. In addition, the concentration of IgA was reported to be reduced in overtrained athletes [41].

A decrease in the concentration of IgA after exercise in endurance sports athletes may be an indication for an increase in the incidence of upper respiratory tract infections [42]. High-intensity exercise decreases the concentration and secretion rate of IgA. However, IgM and IgG concentrations are not reduced [12]. We found a significant difference in IgA and IgM in the SMS group, but there was no significant difference in IgG levels. Gleeson and Pyne [41] found that changes in salivary IgA and IgM can be detected within a few days after high-intensity training but show a negligible effect on IgG levels, suggesting that more than 3 months may be required for significant immunosuppression.

SMS intake decreased ammonia levels during high-intensity exercise, decreased liver function variables (AST and ALT), and altered Ig levels. These results suggest that SMS intake during high-intensity exercise may have a positive effect on fatigue, liver function, and immunity.

The results of this study should be interpreted in the context of the following limitations. First, the participants' daily lifestyle could not be controlled. Second, the number of participants was relatively small and the study period was short. Because this study was limited to male tennis players, it may be difficult to generalize the results to other populations. Therefore, future research will be based on various subjects of other sports, ages, and gender.

## Figures and Tables

**Table 1:** Participant characteristics.

Variables	SMS (*n* = 9)	Placebo (*n* = 9)	*p* value
Age (years)	20.0 ± 1.6	20.0 ± 1.9	0.297
Height (cm)	177.0 ± 4.07	174.9 ± 3.97	0.733
Weight (kg)	69.27 ± 7.07	68.19 ± 5.67	0.608
Muscle mass (kg)	53.90 ± 2.30	52.33 ± 2.98	0.238
BMI (kg/m^2^)	22.11 ± 2.27	22.31 ± 1.57	0.837

Values are presented as mean ± standard deviation. Variables were compared with independent-sample *t*-tests. SMS, *Saengmaeksan*; BMI, body mass index.

## Data Availability

The (DATA TYPE) data used to support the findings of this study are available from the corresponding author upon request.

## References

[1] Jiménez-Reyes P., Pareja-Blanco F., Cuadrado-Peñafiel V., Ortega-Becerra M., Párraga J., González-Badillo J. J. (2019). Jump height loss as an indicator of fatigue during sprint training. *Journal of Sports Sciences*.

[2] Mutch B. J. C., Banister E. W. (1983). Ammonia metabolism in exercise and fatigue: a review. *Medicine & Science in Sports & Exercise*.

[3] Spodaryk K., Szmatlan U., Berger L. (1990). The relationship of plasma ammonia and lactate concentrations to perceived exertion in trained and untrained women. *European Journal of Applied Physiology and Occupational Physiology*.

[4] Wu H.-J., Chen K.-T., Shee B.-W., Chang H.-C., Huang Y.-J., Yang R.-S. (2004). Effects of 24 hr ultra-marathon on biochemical and hematological parameters. *World Journal of Gastroenterology*.

[5] Kwo P. Y., Cohen S. M., Lim J. K. (2017). ACG clinical guideline: evaluation of abnormal liver chemistries. *American Journal of Gastroenterology*.

[6] Praphatsorn P., Thong-Ngam D., Kulaputana O., Klaikeaw N. (2010). Effects of intense exercise on biochemical and histological changes in rat liver and pancreas. *Asian Biomedicine*.

[7] Pal S., Chaki B., Chattopadhyay S., Bandyopadhyay A. (2018). High-intensity exercise induced oxidative stress and skeletal muscle damage in postpubertal boys and girls: a comparative study. *Journal of Strength and Conditioning Research*.

[8] Buckley R. H. (1986). Humoral immunodeficiency. *Clinical Immunology and Immunopathology*.

[9] Dispenzieri A., Gertz M. A., Therneau T. M., Kyle R. A. (2001). Retrospective cohort study of 148 patients with polyclonal gammopathy. *Mayo Clinic Proceedings*.

[10] Pedersen B. K. (2000). Exercise and cytokines. *Immunology and Cell Biology*.

[11] Gonzalez-Quintela A., Alende R., Gude F. (2008). Serum levels of immunoglobulins (IgG, IgA, IgM) in a general adult population and their relationship with alcohol consumption, smoking and common metabolic abnormalities. *Clinical and Experimental Immunology*.

[12] Traeger Mackinnon L., Ginn E., Seymour G. J. (1993). Decreased salivary immunoglobulin a secretion rate after intense interval exercise in elite kayakers. *European Journal of Applied Physiology and Occupational Physiology*.

[13] Venkatraman J. T., Pendergast D. R. (2002). Effect of dietary intake on immune function in athletes. *Sports Medicine*.

[14] Robson-Ansley P. J., Gleeson M., Ansley L. (2009). Fatigue management in the preparation of olympic athletes. *Journal of Sports Sciences*.

[15] Coqueiro A. Y., Rogero M. M., Tirapegui J. (2019). Glutamine as an anti-fatigue amino acid in sports nutrition. *Nutrients*.

[16] National Center for Complementary and Integrative Health Dietary and Herbal Supplements. https://www.nccih.nih.gov/health/dietary-and-herbal-supplements.

[17] Vickers A., Zollman C. (1999). Herbal medicine. *BMJ*.

[18] Lee M. C., Park J. R., Shim J. H., Ahn T. S., Kim B. J. (2015). Effects of traditional chinese herbal medicine Shengmai–san and Pyungwi–san on gastrointestinal motility in mice. *Journal of Korean Medicine for Obesity Research*.

[19] Kwak J. J., Yook J. S., Jeong W. M., Kim J. S., Ha M. S. (2020). Saengmaeg–san as an ergogenic aid: improving exercise performance. *Journal of the Korean Applied Science and Technology*.

[20] Sung Y.-Y., Yuk H. J., Kim D.-S. (2021). Saengmaeksan, a traditional herbal formulation consisting of *Panax ginseng*, ameliorates hyperuricemia by inhibiting xanthine oxidase activity and enhancing urate excretion in rats. *Journal of Ginseng Research*.

[21] Kim H. J., Hong S. K., Min A. Y. (2015). Antioxidant activities and quality characteristics of jelly added with *Saengmaegsan* concentrate. *Journal of the Korean Society of Food Science and Nutrition*.

[22] Lei F., Weckerle C. S., Heinrich M. (2021). Liriopogons (Genera *Ophiopogon* and *Liriope*, Asparagaceae): a critical review of the phytochemical and pharmacological research. *Frontiers in Pharmacology*.

[23] Son I. S., Kim J. H., Sohn H. Y., Son K. H., Kim J.-S., Kwon C.-S. (2007). Antioxidative and hypolipidemic effects of diosgenin, a steroidal saponin of yam (*Dioscorea spp.*), on high–cholesterol fed rats. *Bioscience, Biotechnology, and Biochemistry*.

[24] Lacaille-Dubois M. A. (2005). Bioactive saponins with cancer related and immunomodulatory activity: recent developments. *Studies in Natural Products Chemistry*.

[25] Huang K. C. (1998). *The Pharmacology of Chinese Herbs*.

[26] Attele A. S., Wu J. A., Yuan C. S. (1999). Ginseng pharmacology: multiple constituents and multiple actions. *Biochemical Pharmacology*.

[27] Li Z., He X., Liu F., Wang J., Feng J. (2018). A review of polysaccharides from *Schisandra chinensis* and *Schisandra sphenanthera*: properties, functions and applications. *Carbohydrate Polymers*.

[28] Kwak J.-J., Lee J.-H., Yook J. S., Lee S.-H., Ha M.-S. (2021). Positive effect of *Saengmaeg-san* intake on blood lipid and arteriosclerosis index during high–intensity training. *Journal of the Korean Applied Science and Technology*.

[29] Redondo F. L., Bermudez P., Cocco C. (2003). Evaluation of cobas integra® 800 under simulated routine conditions in six laboratories. *Clinical Chemistry and Laboratory Medicine*.

[30] Bach H. V., Kim J., Myung S. K., Cho Y. A. (2016). Efficacy of ginseng supplements on fatigue and physical performance: a meta-analysis. *Journal of Korean Medical Science*.

[31] Favero T. G., Zable A. C., Colter D., Abramson J. J. (1997). Lactate inhibits Ca^2+^-activated Ca^2+^-channel activity from skeletal muscle sarcoplasmic reticulum. *Journal of Applied Physiology*.

[32] Itoh H., Ohkuwa T. (1991). Ammonia and lactate in the blood after short-term sprint exercise. *European Journal of Applied Physiology and Occupational Physiology*.

[33] Ibanez J., Pullinen T., Gorostiaga E., Postigo A., Mero A. (1995). Blood lactate and ammonia in short-term anaerobic work following induced alkalosis. *The Journal of Sports Medicine and Physical Fitness*.

[34] Chyau C.-C., Ker Y.-B., Chang C.-H. (2014). *Schisandra chinensis* peptidoglycan-assisted transmembrane transport of lignans uniquely altered the pharmacokinetic and pharmacodynamic mechanisms in human HepG2 cell model. *PLOS ONE*.

[35] Huang H., Shen Z., Geng Q., Wu Z., Shi P., Miao X. (2017). Protective effect of *Schisandra chinensis* bee pollen extract on liver and kidney injury induced by cisplatin in rats. *Biomedicine & Pharmacotherapy*.

[36] Gillis C. N. (1997). Panax ginseng pharmacology: a nitric oxide link?. *Biochemical Pharmacology*.

[37] Sun Y., Chen S., Wei R. (2018). Metabolome and gut microbiota variation with long-term intake of *Panax ginseng* extracts on rats. *Food & Function*.

[38] Liu B., Tonkonogy S. L., Sartor R. B. (2011). Antigen-presenting cell production of IL-10 inhibits T-helper 1 and 17 cell responses and suppresses colitis in mice. *Gastroenterology*.

[39] Cooper C. E., Vollaard N. B. J., Choueiri T., Wilson M. T. (2002). Exercise, free radicals and oxidative stress. *Biochemical Society Transactions*.

[40] Levando V. A., Suzdal’nitskii R. S., Pershin B. B., Zykov M. P. (1988). Study of secretory and antiviral immunity in sportsmen. *Sports Medicine, Training and Rehabilitation*.

[41] Gleeson M., Pyne D. B. (2000). Exercise effects on mucosal immunity. *Immunology and Cell Biology*.

[42] MacKinnon L. T., Jenkins D. G. (1993). Decreased salivary immunoglobulins after intense interval exercise before and after training. *Medicine & Science in Sports & Exercise*.

